# Effects of family planning on fertility behaviour across the demographic transition

**DOI:** 10.1038/s41598-021-86180-8

**Published:** 2021-04-23

**Authors:** Karen L. Kramer, Joe Hackman, Ryan Schacht, Helen E. Davis

**Affiliations:** 1 Department of Anthropology, University of Utah, Salt Lake City, UT USA; 2 Department of Anthropology, East Carolina University, Greenville, NC USA; 3 Department of Human Evolutionary Biology, Harvard University, Cambridge, MA USA

**Keywords:** Ecology, Evolution, Psychology, Health care

## Abstract

The adoption of contraception often coincides with market integration and has transformative effects on fertility behavior. Yet many parents in small-scale societies make decisions about whether and when to adopt family planning in an environment where the payoffs to  have smaller families are uncertain. Here we track the fertility of Maya women across 90 years, spanning the transition from natural to contracepting fertility. We first situate the uncertainty in which fertility decisions are made and model how childbearing behaviors respond. We find that contraception, a key factor in cultural transmission models of fertility decline, initially has little effect on family size as women appear to hedge their bets and adopt fertility control only at the end of their reproductive careers. Family planning is, however, associated with the spread of lower fertility in later cohorts. Distinguishing influences on the origin versus spread of a behaviour provides valuable insight into causal factors shaping individual and normative changes in fertility.

## Introduction

The trend toward smaller families, ongoing today in small-scale societies worldwide, is broadly linked to market integration^1–5^. However, the emphasis on declining average family size can mask underlying changes in the structure of fertility within a population^6^. The distribution of fertility across the transition has been described as moving from relatively low^7,8^ to high variance and then stabilizing back to lower levels^9^. Unknown, however, is how this plays out longitudinally or within a population. Under natural fertility (absence of parity-specific fertility control) ^10^ ^11^ family size within a population varies largely due to stochastic individual differences in fecundity^12^. What follows with the onset of the fertility transition is of interest because parents diversify their childbearing strategies^13^, with some having much smaller and others larger families than in previous cohorts. This change in the distribution of fertility gives new norms a chance to emerge. While the elevation of fertility variance has been described at the population level, to our knowledge the mechanisms motivating it are unidentified, yet are crucial to understand how new family size norms take hold in a population and provide insight into factors affecting the origin versus spread of declining fertility^14^.

### Uncertainty in family planning

In small-scale societies with no prior experience with the labor market economy, uncertainty (the likelihood of a particular outcome, either its success or failure, is unknown^15^) often accompanies market integration (the transition from a  local subsistence economy to greater involvement in the regional and national economy). It is in this context that parents are also exposed to new fertility options. Under premarket, natural fertility conditions, fertility is correlated with traditional measures of wealth (the food, resources, and help available to a mother) due to their physiological correlation with energy balance and fecundity^12,16–22^. While cultural factors also mediate family size on both sides of the transition, through for example, norms about sexual access, exposure to modernizing influences affects energy balance and constraints on fertility^19,23,24^. The introduction of calorie-rich market foods, mechanized farming, water pumps and other domestic appliances relaxes physiological constraints on female fecundity. At the same time, modernizing influences open up new options to reduce family size and invest in child quality^13,25,26^.

If incentives for both larger and smaller families coexist, and the costs and benefits to adjust family size are uncertain, the path forward in terms of fertility decisions may be unclear. Some parents may continue to depend on known norms and rules of thumb, using what they know about the number of children that they can support given their traditional livelihoods. In this scenario, fertility may increase as maternal energetic and physiological constraints are eased, a demographic trend reported in many small-scale societies during the first stages of market development^19,27–30^. However, other parents may be less risk adverse, forego their children’s domestic, field, or foraging labor, have fewer children, find novel ways to generate capital, and send their children to school or otherwise prepare them for wage work. Ultimately, *when fertility payoffs are uncertain*^6^, we suggest that *individuals vary in their reliance on established versus newly emerging behavioural rules and pursue diverse childbearing strategies* such that fertility variance is observed to increase at the population level.

The uncertainty introduced by market development may be alleviated by uncovering payoff structures through social learning and exposure to new ideas . An individual may emulate the behaviours of others to short-cut the time required to learn about potential costs and benefits of having smaller families. Propinquity helps to explain, for example, why even after controlling for structural factors, fertility decline occurs more rapidly in densely populated urban areas than in isolated rural areas^31–35^. Social learning effects on fertility decline has a large and productive literature, including both diffusion^4,36–44^ and cultural transmission^14,45–47^ perspectives. (For economic approaches to global trends toward smaller families see^1–5,48–55^.) For example, when high status or prestigious individuals adopt new ideas or innovations that limit fertility, others are likely to imitate these behaviours, resulting in a shift in cultural norms toward lower fertility preferences^45^. Diffusion and cultural transmission approaches share in common an emphasis on changes in the ideal number of children, the perceived value of children, and norms regarding reproductive behavior^33,56–58^.

### Cultural transmission and fertility-limiting family planning

The adoption of fertility-limiting family planning is a widely used proxy measure of cultural transmission in the spread of low-fertility norms^37,47,59–61^. Debate persists whether cultural transmission or economic influences offer competing explanations or are codrivers of the fertility transition^33,56–58^, and the extent to which cultural transmission models explain the origin versus the spread and maintenance of low fertility^14^. To further this discussion, we track the role of social learning across the transition to differentiate the effects that the adoption versus spread of family planning have on fertility decline within a small-scale society.

We use longitudinal individual- and household-level fertility, social network and economic data for indigenous rural Maya women living in Campeche, Mexico to first characterize the distribution of fertility across the transition, and determine whether within-population variance precedes a change to low fertility. While the rise in variance has been described with cross-sectional data for populations already amidst the transition^9^, and in population-level comparisons^7^, empirical longitudinal data that span natural to contracepting fertility are needed to demonstrate that this is indeed a within-population characteristic of fertility transitions. To characterize variance across the fertility transition, we use known determinants of fertility (wealth, household size, wage-labor engagement and education) to model changes in childbearing behavior. We then model the influence that family planning has in driving changes in the distribution of fertility. Our community census data include women who were at risk of conception from 1932 to 2018, a time period that brackets the transition from a natural fertility to a contracepting population, and from an egalitarian subsistence farming economy to a mixed-market economy. A paved road, which linked this remote, rural community to larger market towns^62^, was built in the early 2000s and initiated sweeping economic changes including the adoption of family planning. This longitudinal perspective leveraging  detailed reproductive histories and household economic data allow us to detect factors underlying changes in fertility behavior and to distinguish the influence that family planning has on the origin versus the spread of low fertility.

## Results

### Characterizing the distribution of fertility across the transition

**Figure 1:**
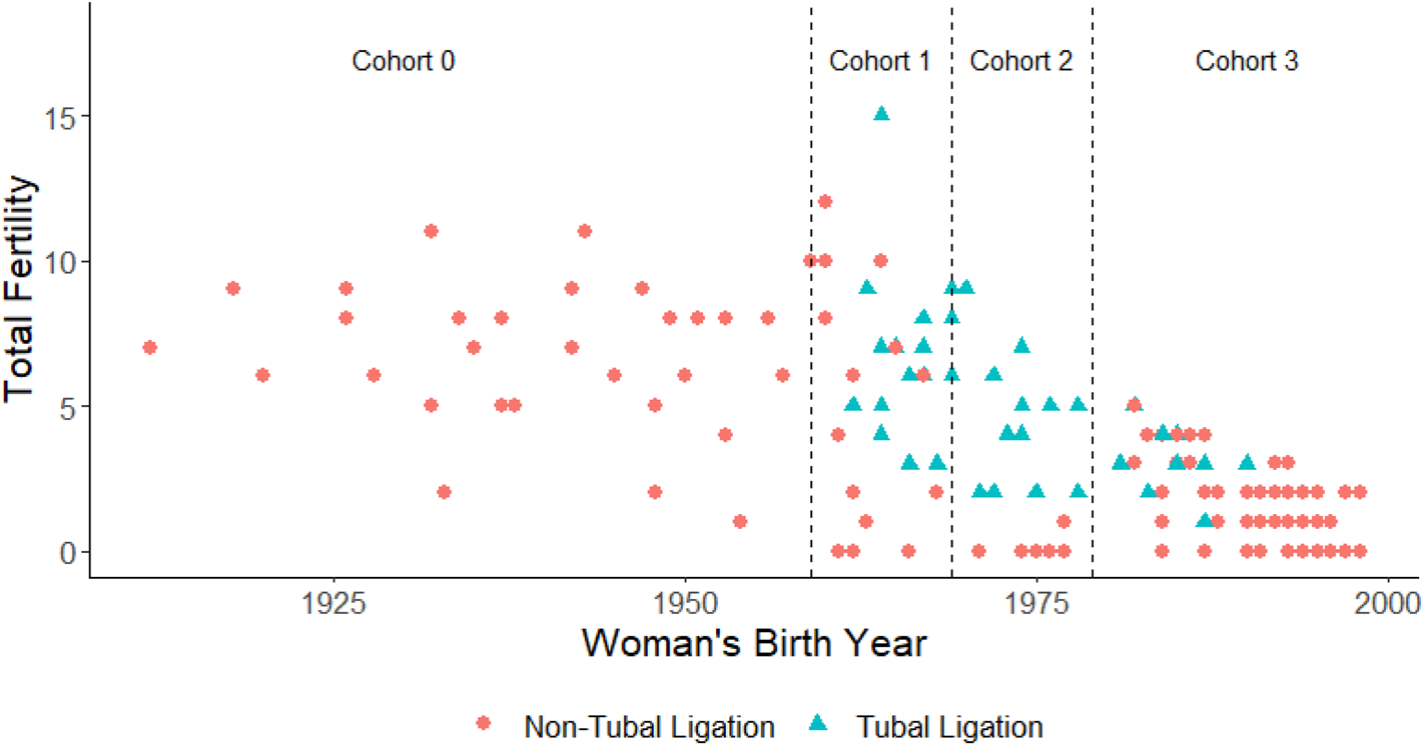
Total fertility across time stratified by women who have (triangles) and have not had a tubal ligation (circles). Dashed lines indicate cohort boundaries. Completed fertility (women > 40 years old) is shown for Cohorts 0–2. Women in Cohort 3 are still of reproductive age (ages 20–39) and values reflect total current fertility (number of live births through 2017).

To assess how the distribution of fertility has changed over time, our census data includes Maya women born from 1912–1999 (n = 161) and their 600 children (birth years 1927–2018). Women were subset into four cohorts (Fig. 1; Table 1). The first cohort of women was born and completed their entire reproductive careers before the introduction of the road and modernizing influences. This cohort (Cohort 0) serves as a natural fertility baseline. Following the introduction of the road, we stratify women who completed their reproductive careers into two 10-year cohorts (Cohorts 1–2). We also include the current cohort of women, ages 20–39, who have yet to complete their reproductive careers (Cohort 3).

**Table 1:** Cohort  descriptive characteristics.

	Pre-road		Post-road	
	Cohort 0	Cohort 1	Cohort 2	Cohort 3
Birth years	< 1959	1960–1969	1970–1979	1980–1999
Age at road introduction	>40	30–39	20–29	10–19
Current Fertility Status	Completed	Completed	Completed	Ongoing
Year data collected	1993	2013	2017	2017
N	31	32	22	76

**Figure 2:**
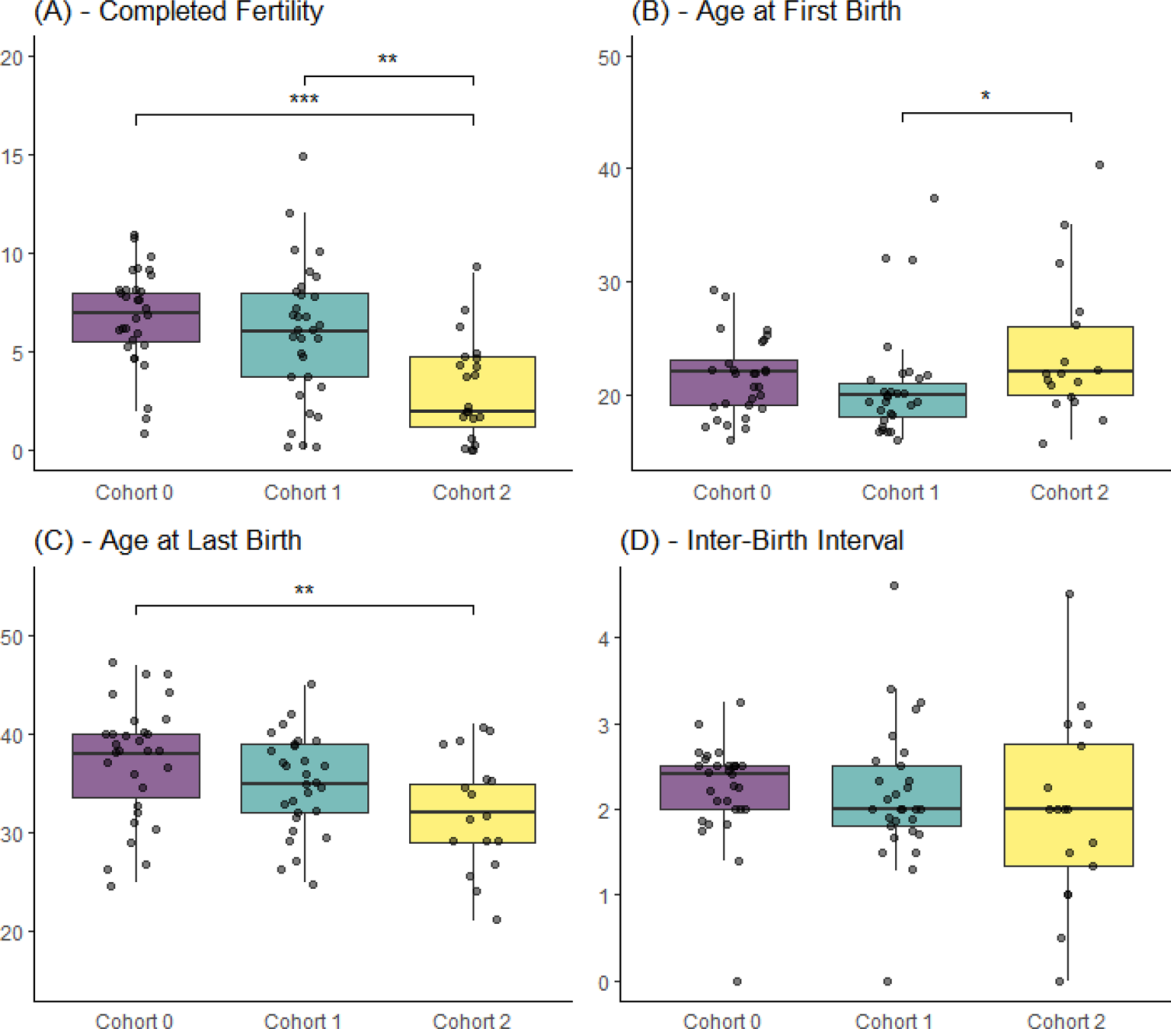
Reproductive traits by cohorts with completed fertility. Pair-wise comparisons shown only for significant results for mean differences, using Wilcox sign test *p <  = 0.05; **p <  = 0.01; ***p <  = 0.001 (significance tests presented in Supplementary Results Table 2). Boxplots display the mean with the upper and lower quartiles, with whiskers showing the 95% CI around the mean estimate.

We first establish whether an increase in variance proceeds a significant decline in fertility and find that in the first decade after the introduction of the road (Cohort 1), mean completed fertility does not change in a statistically significant way (although it is lower) and appears to have stalled at high levels (mean completed fertility in C0 = 6.8; C1 = 5.9, *p* = 0.18). However, the variance significantly increases (CV_C0_ = 0.36 vs. CV _C1_ = 0.59; *p* = 0.02). The distribution of completed fertility in Cohort 1 expands both at the upper end as some women have larger families, and at the lower end as other women have much smaller families compared to the baseline cohort (Fig. 2A; Table 2; Supplementary Fig. 1). Two decades after the introduction of modernizing influences, family size then significantly declines to 3.0 children (*p* < 0.001; Supplementary Table 2). While the raw variance begins to decline, the variance relative to the mean continues to increase (CV_C0_ = 0.36 vs. CV _C2_ = 0.84, *p* < 0.0001) compared to the baseline pre-road cohort. Thus, the first cohort following the completion of the road shows a marked increase in variance with no significant change in mean fertility, which only becomes evident two decades later.

**Table 2:** Descriptive statistics of model variables by cohort.

	Cohort 0	Cohort 1	Cohort 2	Cohort 3
	Mean (SD)	Mean (SD)	Mean (SD)	Mean (SD)
**Demographic variables**				
Age at first birth	21.2 (3.7)	20.8 (4.9)	23.8 (6.4)	20.8 (3.2)
Age at last birth	37.3 (5.9)	35 (4.9)	32.1 (5.9)	25.5 (3.6)
Total fertility	6.8 (2.5)	5.9 (3.5)	3.0 (2.5)	1.7 (1.5)
Total fertility-coefficient of variation	0.36	0.59	0.84	0.87
**Family planning variables**				
Tubal ligations N (%)	0 (0%)	17 (53%)	16 (73%)	12 (16%)
Age at TL	NA	36.4 (5.6)	32.1 (5.2)	24.7 (2.2)
**Traditional determinants**				
Hectares under cultivation	2.9 (1)	5.8 (3)	7.1 (3.3)	9.1 (8.8)
Hectares Gini	0.20	0.27	0.26	0.46
Sharing group size	4.3 (1.6)	5.5 (2.7)	4.6 (2)	4.9 (2.6)
Sharing group Gini	0.21	0.27	0.24	0.29
**Novel determinants**				
Years of education	1.3 (1.3)	3.0 (2.1)	3.9 (2)	9.4 (2.6)
Proportion of Wage-laborers	0.2 (0.2)	0.1 (0.2)	0.2 (0.2)	0.3 (0.2)

Total fertility is defined as the total number of live births by a woman in the year 2017. For Cohort 0, 1, and 2, total fertility is equivalent to Completed Fertility. Descriptive accounts of variable creation are presented in Supplementary Methods.

### How determinants of fertility shift across the fertility transition

Two sets of fertility determinants are included in models to evaluate changes in their influence across the fertility transition. The first set are traditional determinants, which are parameterized as *land* (number of hectares under cultivation; see Supplementary Methods Variable Construction) and *sharing-group size* (the number of adults over the age of 15 in a sharing group, a grouping similar to a household). Land under cultivation was determined both through economic interviews and accompanying farmers to their fields and taking GPS measurements of the perimeters their cultivated fields. Sharing groups were identified through detailed social network and economic interviews, and define groupings of biological families that are observed and self-identify as sharing food, labor, capital and other resources. Because sharing group is independent of biological family size, a decline in fertility over the four cohorts may not affect sharing group size ^64^ (see Table 2) .

The second set are novel determinants parameterized as *education* (a mother’s completed years of schooling) and *wage labor* (a binary variable indicating whether 50% or more of the adult members of the household engage in wage-labor). Since most households engaged in some form of occasional or part-time paid labor after the construction of the road and because the amount of time each laborer spends is quite variable, we chose to use a dummy variable, which indicates whether a household has a particularly strong commitment to wage-labor. (Supplemental analyses with alternative variables defining wage-labor status are given in Supplementary Tables 4 & 5.) Both education and wage labor participation are proxies for a household’s engagement in the market economy, and are well-documented predictors associated with fertility reduction cross-culturally^63–65^.

**Figure 3:**
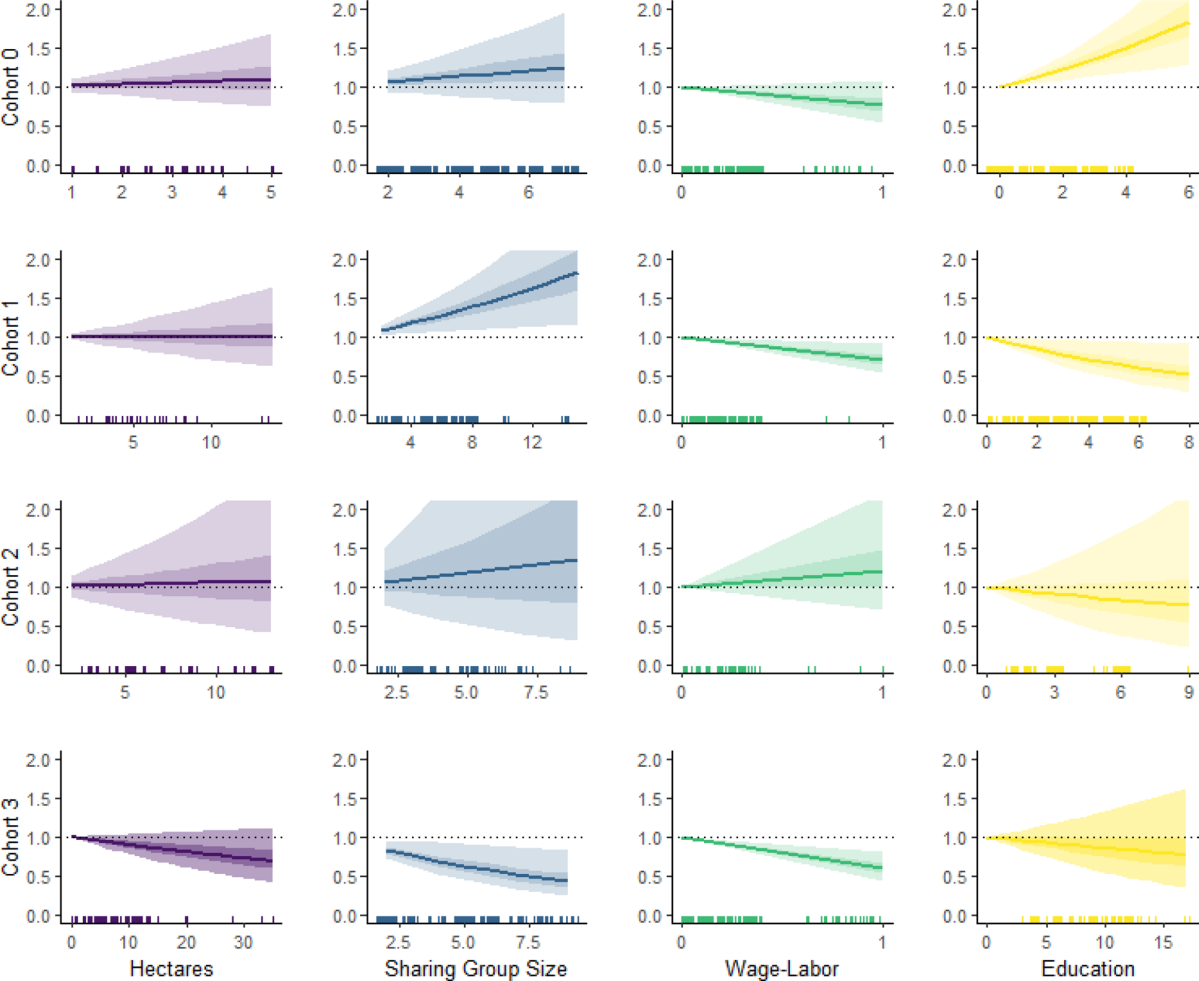
Adjusted hazard ratios. Lines reflect the % increase in the yearly probability of giving birth compared to baseline for a given increase in the covariate. The baseline reflects the hazard rate for women at a value of 0 for the covariate. Shaded areas reflect the 50% and 95% of the simulated distribution of coefficients^66^. Statistically significant effects do not overlap with the dotted line (also see Table 4). Raw data plotted on x-axis have been jittered, and are on different scales. *Note* that the positive association between education and fertility in Cohort 0 is likely not meaningful as an exception to the robust cross-cultural finding that education and fertility are usually negatively associated. In Cohort 0, completed education averages 1.3 years, with less than 4 years as the maximum schooling for any girl (Table 2). Given the low levels and young age at completed education, girls, if they did go to school, would not have faced trade-offs between continuing schooling and starting a family.

Results of a Cox proportional hazards model give the estimated hazard ratio, or the percent increase or decrease in the probability of a birth occurring compared to women at baseline, which is the probability of giving birth when the value of the explanatory variables is at 0 (Fig. 3; Table 3). In the first decade after the introduction of the road and modernizing influences (Cohort 1), positive effects of sharing-group size emerge alongside negative effects of both education and wage-labor. In Cohort 2, the model showed a poor fit to the data, and none of the explanatory variables had significant effects on the yearly probability of giving birth. In Cohort 3, which includes women who are in the midst of their reproductive careers, education and household participation in wage labor have continued negative effects on the probability of giving birth, while effects of sharing-group size shift from positive to negative.

**Table 3:** Hazard ratios (95% CI).

	Cohort 0	Cohort 1	Cohort 2	Cohort 3
Hectares	1.02 (0.94, 1.11)	0.99 (0.96, 1.03)	1.01 (0.95, 1.08)	1.00 (1.00, 1.02)
Sharing group size	1.03 (0.97, 1.10)	1.04** (1.01, 1.06)	1.04 (0.88, 1.23)	0.89***(0.83, 0.97)
Years of education	1.11** (1.04, 1.18)	0.92* (0.86, 0.99)	0.94 (0.82, 1.08)	0.98 (0.90, 0.98)
Wage-labor	0.76 (0.53, 1.10)	0.66*** (0.53, 0.83)	1.11 (0.58, 2.12)	0.59***(0.46, 0.95)
Tubal ligation	NA	1.04 (0.86, 1.25)	NA	1.35** (1.13, 1.74)
Observations	235	205	87	199
Wald test	18.81*** (df = 4)	267.14*** (df = 5)	1.28 (df = 4)	35.53*** (df = 5)

Bold indicates statistically significant effects. *p < 0.05 **p < 0.01 ***p < 0.001.

In sum, in the first cohort after the road, fertility is positively associated with traditional determinants of fertility, particularly sharing-group size, a proxy for the availability of maternal labor support. At the same time, novel determinants (wage labor and education) begin to have negative effects. At the population level, the combination of these contrasting influences on the probability of giving birth reflects a diversification of childbearing strategies and underlies the substantial increase in fertility variance in Cohort 1.

### Does family planning drive changes in distribution of fertility across the transition?

As elsewhere in Latin America, the introduction of fertility-limiting family planning in the Maya community took the form of tubal ligation. Female sterilization is the most widely used contraceptive method in developing countries^67^, and the prominent method in Latin America, with sharp increases in prevalence through the early 2000s^68^. In Mexico, tubal ligation is the most common form of fertility control, utilized among 58% of contracepting married women^69^, 2006 data, Tables 1 & 2. For local Yucatec Maya women, tubal ligations became available as a consequence of hospital births, which were uncommon prior to a paved road being built in 2000. After delivery, women are offered, and often encouraged to have the procedure. Hospitals are located in larger towns some distance from the study area, and women often stayed with relatives in those towns toward the end of their pregnancies and after they were discharged from the hospital prior to traveling home. Birth, hospital experiences and procedures are widely discussed among women and it is common knowledge among female community members who has had a tubal ligation. A few women (5) in recent years report using other forms of contraception, but these were unknown to  women in Cohorts 1 and 2.

If the increase in fertility variance early in the transition is driven by the adoption of family planning, in this case by abruptly shortening the reproductive window, we expect to see a significant effect on the probability of giving birth in Cohort 1. In other words, the question we address is *do early adopters of family planning drive the lower bound of family size down below what is feasible under natural fertility conditions, thereby increasing fertility variance?*

Although the trend to have a tubal ligation (TL) increases over time (see Table 2), when in their reproductive careers women have a TL, and its effect on fertility variance, varies across the transition. Model results show that TL does not have a significant effect on the yearly probability of giving birth in the first cohort after the road was built and modernizing influences were introduced. Women in Cohort 1 received a TL after they have had six to seven children (mean = 6.8; SD = 2.8), and at higher parities than those who do not. Having a TL appears initially to reflect the decision not to go on to have families of seven or more children, and to maintain an upper limit of fertility consistent with women’s preferences at baseline. Other reproductive traits do not significantly differ between women who elect to have a TL and those who do not (Table 4; see Fig. 2). Thus in answer to our question, while a TL curtails the potential to have eight or nine children, its adoption is not initially associated with driving up the variance or lowering fertility.

**Table 4:** Comparison of Women with and without tubal ligations.

	Cohort 1			Cohort 3		
	Non-tubal ligation (N = 12)	Tubal ligation (N = 17)	*p* value	Non-Tubal ligation (N = 41)	Tubal ligation (N = 12)	*p* value
**Demographic traits**						
Age at first birth	22.1 (6.1)	19.9 (3.7)	0.28	21 (3.2)	20 (3.2)	0.34
Age at last birth	34.0 (4.8)	35.6 (5)	0.38	25.3 (4)	26.1 (2.3)	0.38
Completed fertility	4.9 (4)	6.8 (2.8)	0.14	**2.3 (1.1)**	**3.2 (1)**	**0.02**
Age	–	–		**27.3 (4.5)**	**31.7 (2.5)**	** < 0.001**
**Socioeconomic**						
Hectares of land	6.2 (3.2)	5.3 (2.8)	0.43	**8.3 (8.1)**	**5.2 (3)**	**0.05**
Years of education	2.5 (1.8)	3.4 (2.3)	0.21	**9.4 (2.6)**	**7.9 (1.7)**	**0.03**
Sharing group size	5.2 (2.3)	5.7 (3.1)	0.60	4.5 (2.6)	4.4 (2.4)	0.90
Proportion of wage labor	0.1 (0.2)	0.1 (0.2)	0.90	0.2 (0.2)	0.3 (0.2)	0.40

Table does not include comparisons for Cohort 2 because 16 out of 17 women who had children received a Tubal Ligation. Comparisons excludes nulliparous women since they do not tubal ligations.

Family planning strategies shift by Cohort 2, where most women received a TL, and had them at earlier parities, after 3–5 children. This trend of having a TL at earlier parities continues into Cohort 3. The average completed fertility of women in Cohort 2 who had TL (N = 16, 62%) was 4.1 (SD = 2.1), and 3.2 (SD = 1.1) for women in Cohort 3. Clearly, in later cohorts a TL substantially shortens women’s average reproductive careers by significantly lowering age at last birth (Table 4; see Fig. 2), and is associated with having fewer total children. Within Cohort 3, women who have a TL have higher fertility than those who do not (3.2 vs. 2.3 children, Table 4), and are on average 4 years older. We note that this is not contrary to the long-term trend toward lower fertility, e.g. since women in Cohort 3 are still of reproductive age we do not yet know how the completed fertility of TL and non-TL women will compare. Those who have not had a TL are younger than those who have and potentially will go on to have more children.

In sum, for the early adopters, TL is not associated with a decline in completed family size, or other reproductive traits. Nor does the uptake of fertility-limiting family planning appear to drive the increase in variance. It takes another decade to see a substantial effect of TL on fertility.

## Discussion

The fertility transition has been extensively described, with a focus on regional, national, and global trends to smaller average family sizes. Much less attention has been paid to factors underlying changes in the distribution of fertility within a population^6,70^. Our longitudinal data span the transition from a natural fertility, equalitarian, subsistence society to a market-integrated, contracepting society and provide insight into the factors motivating changes in the distribution of fertility. Prior to market integration, Maya women had approximately equal access to the land and labor needed to raise children (Cohort 0 Gini Coefficients: Hectares of land under cultivation = 0.20, Sharing group size = 0.21, Completed fertility = 0.19; Table 1 & Supplementary Methods Extended Sample Description). Our baseline starts with a relatively homogenous population, which was historically shaped by the *ejido* land tenure system and lack of proximity to markets. Since its institution following the Mexican Revolution, the *ejido* system stipulates the collective ownership of agricultural lands, which cannot be bought, sold or inherited. While the road exposed the study community as a whole to a new suite of opportunities and information, individuals made choices how to respond. The natural fertility baseline, and what follows, offers a rare vantage point to view the role of contraception in fertility decline across the transition, and changes in the structure of fertility within a population.

### Family planning’s role in the origin vs. spread of low fertility

As a well-established metric for the influence of cultural transmission on low fertility norms^37,59–61^, we track the uptake of family planning across the fertility transition to differentiate the effects that its adoption versus spread has on fertility. Following market integration, which coincides with the introduction of family planning, fertility variance significantly increases *before* changes in average family size can be detected. Model results show that the dramatic increase in variance is the outcome of the contrasting influences that traditional and novel determinants of fertility have on family size, an effect noted in other cultural contexts^13^, rather than of the adoption of family planning. Market engagement, the availability of new foods and the introduction of mechanization attenuate physiological constraints on fertility^19,71^. Under these circumstances, some Maya parents appear to rely on what they already know about the number of children they can support with maize agriculture and household labor, and continue to have large, and in some cases, even larger families. Indeed, subsistence populations undergoing market integration exhibit some of the highest fertility documented^19,27–30^. At the same time, other parents have fewer children. Ultimately, the contrasting effects of traditional wealth measures being associated with higher fertility and novel determinants being associated with lower fertility result in little change in average family size, but a substantial change in variance in Cohort 1. To the latter point, our longitudinal analysis confirms the cross-sectional observation that fertility moves from a Poisson distribution (where the mean and variance are approximately equal) to an over-dispersed Poisson (where the variance becomes signifiantly greater than the mean) after the onset of the transition^9^. Further, our analyses provide evidence that increased variance is a characteristic of the fertility transition observable both as fertility rates across different populations^7^ as well as within populations .

While this pattern has been described, the trigger mechanisms have remained unidentified. We find that family planning was adopted as it became available with market integration. But initially it has little influence on fertility variance, lowering total fertility or distinguishing the reproductive traits of those women who received a tubal ligation and those who did not. For the first cohort of women, family planning serves to maintain the upper limit of fertility, rather than pushing down the lower bound of fertility. This finding is consistent, for example, with studies among rural Ethiopian women^72^ and Gambian women^73^, who are more likely to adopt contraception at higher than lower parities. The relationship between contraception and fertility is complex in that it depends whether the population is viewed longitudinally across cohorts or within cohorts. Longitudinally, lower fertility is associated with contraceptive use, while within cohorts it may not be; higher than desired fertility may in fact be what motivates women to adopt contraception. Because Cohort 3 women have not yet completed their reproductive careers, younger women may go on to have another child before committing to a TL, and the difference in fertility between the two groups likely reflects age differences and comparing completed fertility (women who have had a TL) with  ongoing fertility (non-TL women).

While the adoption of family planning does not appear to explain the origin of fertility decline, it contributes to the *spread* of lower fertility later in the transition. Exposure to outside social influences and learning increase over time with market integration, and by Cohort 2 nearly all Maya women who completed fertility had tubal ligations, which then has a clear effect on lowering fertility through earlier stopping behaviors. The lag time between the adoption of family planning in Cohort 1 and its effect on reproduction in Cohort 3 suggests that family planning itself does not instigate a change in fertility norms. The extent that the uptake of contraception represents cultural transmission^44,73^, this finding has implications for the debate concerning whether social versus economic influences explain fertility decline. While our model is not designed to evaluate economic influences per se, the uptake of family planning, as an indicator of social learning, is not initially associated with fertility decline.

### Origin of the fertility variance as the response to uncertainty

While initial exposure to new childbearing options and market opportunities was relatively homogenous, we expect individual responses to vary. The two-decade delay between the adoption of family planning and evidence of a fertility decline, we propose reflects the uncertainty of parents to commit to smaller families until they gain sufficient knowledge to weigh the costs and benefits of having fewer children. On a novel fitness landscape, given that the payoff to have either small or large families is yet unknown, we suggest that the response to this uncertainty is diversification of childbearing strategies at the population level, e.g. some parents double  down on what they know and have  the same or more children, while others take advantage of new investments in child quality and have fewer children.

Variance in reproductive and behavioral strategies as a response to uncertainty has been studied nonhuman animals^74,75^. For example, environmental uncertainty can favor bet-hedging in reproductive strategies whereby parents vary their investments and reproductive output in attempts to match offspring phenotypes with alternative future environments^76,77^. Some evidence shows that when resource constraints on reproduction are diminished, as occurs when animals are relocated from wild environments to captivity, they exhibit unexpected increases in morphological variability^78^. Models of human fertility also have connected uncertainty and reproductive responses. When unpredictability is high or environmental circumstances calamitous, models predict both over-reproduction^6,70^ and low fertility^25,79,80^. Our empirical results suggest that during environmental fluctuation, both high and low fertility (e.g. increased variance within a population) are responses to environmental uncertainty.

When the costs and benefits to family size adjustments are unknown, we propose that parents diversified childbearing strategies. Prior to market integration, parents know the value of goods and services and can make informed decisions about the number of children to have based on their anticipated food requirements, labor and production needs, and goals for child success. With market integration, in the Maya case instigated by a paved road, this known payoff structure is replaced with uncertainty such that parents cannot estimate the cost that smaller families may have on reducing the family work force or the benefit it may have for their children to pursue schooling and new economic opportunities. For instance, parents in the Dominican Republic who have no experience with formal education lack the prior knowledge to weigh the benefits to school their children versus the costs to relinquish their economic production^81^. We suggest that this situation of uncertain payoffs characterizes the origin of many fertility transitions, and is the one that many small-scale societies find themselves in now, and has played out repeatedly over human history when technological innovations introduce new costs and benefits to family size decisions^6,70^.

Different strategies may begin to emerge and then be perpetuated as some families gain access to market wealth, education or other new forms of status. Our results show that fertility tends to decline faster in those Maya households where wage-labor has become the dominant livelihood strategy (50% or more of the adults engage in some form of wage labor), than in mixed-economy households engaged in both agriculture and wage labor. Although new reproductive norms have yet to coalesce (e.g. variance is still high), women in predominantly wage labor households had the lowest yearly probability of giving birth. The trend toward smaller families, thus, is nuanced. While average family size is declining at the population level, it begs the future question whether divergent childbearing strategies will persist such that different fertility norms will form around distinct economic strategies.

Finally, our cohort analysis captures the well-known reversal of wealth effects on fertility, where wealth and status become decoupled from fertility^13,82–84^. Over the course of four cohorts of Maya women, sharing-group size, land wealth and education all move from having relatively positive effects on fertility to having negative influences on fertility behaviors.

## Conclusions

In small indigenous communities world-wide, market integration can instigate transformative changes in fertility behavior. The Maya data show that during the early stages of market integration, before mean changes in fertility are evident, a diversification of childbearing strategies appears to be a first response to a new, yet uncertain environment. At the community-level, before the effects of family planning takes hold, childbearing strategies transition from being relatively homogenous to markedly heterogeneous. We highlight uncertainty as a key mechanism motivating the increase in variance, which results from the contrasting negative effects of novel determinants (education, wage labor) pushing down fertility for some, and the positive effects of traditional determinants (land & labor pool) raising fertility for others. While the swing from low to high variance may settle down in the future, an important implication is that increased variance gives the foothold for inequality to emerge, and fertility to coalesce around different norms as parents pursue divergent childbearing strategies.

## Methods

### The Yucatec Maya study population

The Maya study population is located in the Puuc region of the central Yucatan Peninsula, Mexico. Multigenerational economic, demographic, and social network data have been collected in this community since the early 1990s^19,85–87^. Prior to 2000, all community members were subsistence maize farmers and the household was the unit of production and consumption. Because of the lack of roads and vehicles, little means existed to engage in cash cropping, formal schooling, wage-labor, or the regional economy. Wealth was measured by maize production, and high fertility was an asset because the size of the household labor force was directly related to the amount of maize that could be produced^88^.

A paved road was built into the community in the early 2000s, which introduced access to mechanized farming and means to transport crops to market, children to schools, and adults to wage labor jobs. However, costs and benefits to these new opportunities were clouded with uncertainty. Many households intensified maize production, putting more land under cultivation (see Table 2), and although the Maya have been successful farmers for millennia, the potential payoffs to agricultural intensification involved novel market forces, price volatility, and, importantly, debt. Many new subsistence practices were also introduced by the government, NGOs and commercial agencies. Some succeeded, but many others failed. For example, vegetable gardening, nut orchards and cattle herding all failed as new commercial ventures, while honey production is currently thriving.

With market access, parents also faced new decisions affecting the demand for children—whether to send them to school and forgo their time and labor contributions to the household, whether to leverage debt and invest in mechanized farming, or to become a wage laborer, abandon farming and commit to buying food. Parents have clearly articulated this changing and complicated economic and fertility environment. They will, unprompted, describe the quantity-quality tradeoff—if they have fewer children, they could afford to send more of them to school. But, parents also express uncertainty whether schooling will have a social or economic benefit for children or be a net cost because they will lose their agricultural labor base and children will lose their place in the cue for land tenure status.

While the road and market access offered many new opportunities, these changes also altered the landscape of decision making for Maya families. Parents, who previously could anticipate the number of children they could support, given the known relationship between family labor and maize production, now must consider the trade-offs to  invest in children’s schooling, and other forms of human capital beneficial in a wage-labor economy, relative to the loss of household labor. Other aspects of Maya social life remain similar today as they were in the past. The community continues to be part of a largely rural, underpopulated region that is ethnically homogeneous. Although migration for marriage, and occasionally work, occurs, marriages are overwhelmingly endogamous, with most women forming unions with other community members. No internet is available, and few villagers own phones, televisions, or computers.

### The sample of Maya women

Demographic variables were taken from annual reproductive histories collected from all women alive in or born since 1992 (n = 161), the year of initial data collection. Relevant variables include age at first birth, age at last birth, year of each live birth, interbirth interval, total fertility, and year women had a tubal ligation. We recognize that a decline in infant mortality may lead to an increase in the number of surviving offspring, affecting changes in the variance. Infant mortality has been low in rural Yucatan throughout much of the historic period (water-born and other infectious disease insults are low in this rural region in large part because the population is dispersed, adequately fed and obtain water from closed underground water sources), and over the 26-year study period no significant changes in infant mortality have been documented in the community^62,89,90^.

All women age 15 and older are included in the analyses except those who emigrated and censused out of annual reproductive surveys (*n* = 3), who were very elderly in 1992 and their reproductive histories could not be validated in later censuses (*n* = 2), or who had known fertility issues (e.g. no child born after 5 years of marriage; *n* = 4; Supplementary Table 6). No other exclusion criteria were used.

Women were subset into four cohorts (Fig. 1, Table 1) as described in the main text. 10-year cohorts balance capturing temporal dynamics in reproductive behavior that would be obscured if the cohort range was expanded, and limiting sample sizes and lowering the power statistically detect patterning to if the cohort range were shorter.

Informed consent was obtained from all subjects or, if subjects are under 18, from a parent and/or legal guardian, and all methods were carried out in accordance with relevant guidelines and regulations.

### Characterizing changes in the distribution of fertility across the fertility transition

To determine whether an increase in variance proceeds a significant decline in mean fertility, we use an asymptotic test for homogeneity of variances using the R package cvequality^91,92^. The coefficient of variation is calculated as the standard deviation divided by the mean and reflects the variance scaled to the mean of the distribution. To account for small samples, we also test for differences in variance using a bootstrapped homogeneity of variance test on the unscaled variances^93^, which produce equivalent results to the coefficient of variance (Supplementary Sect. 1e). We test for significant differences in mean fertility across Cohorts 0–2 using Dunn’s Kruskal–Wallis nonparametric pairwise multiple comparison procedure, with a conservative post-hoc test that adjusts the *p* values for multiple testing.

### Modeling changes in determinants of fertility across the fertility transition

To model the influence that traditional (land, and sharing group size) and novel (education and wage labor) fertility determinants have on changes in the distribution of fertility across the transition, we employ a recurrent event analyses, using an independent Cox proportional hazard model for each of the four cohorts. To account for correlated observations (births) within women, we employ a robust semiparametric analysis using a cluster id for women in each cohort using the ‘Survival’ package in R^94^. Recurrent event analysis permits modelling the full birth history of each woman within a cohort to estimate the effects of the covariates on the hazard rate, or the yearly probability of giving birth for each cohort (n = 3077 risk years). This approach offers several advantages to analyzing aggregate counts (e.g. births per woman). First, recurrent event analysis increases the power to detect effects by drawing on information about the timing of births across a woman’s reproductive career, rather than simply her total fertility. Second, recurrent event analysis permits estimating covariate effects that are comparable for women who both have completed their fertility (Cohorts 0–2) and who are still in their reproductive careers (Cohort 3), which allows us to make inferences about current fertility trends.

Women census into the model at age 15 and reach a terminal event when they are no longer at risk of birth, either at age 50 or when they have a tubal ligation. *Total fertility* is summarized as the sum of live births a woman has had up to the year 2017, the last year of the survey. For women in Cohorts 0–2, this represent *completed fertility*, because all women have reached a terminal event. Because women in Cohort 3 are still of reproductive age (ages 20–39), they are right censored at end of survey in 2017. While fertility data have been collected annually since 1992, model covariates were collected at three time points, in 1993, 2013 and 2017; descriptive statistics of each covariate by cohort are given in Table 2. All predictors included in the models thus are time-invariant, as they are collected at three timepoints.

Nulliparious women are included in the recurrent event history analyses in modeling changes in determinants of fertility. They were not included in modeling family planning influences (described below) since only women who have given birth have tubal ligations. Sensitivity analyses show that the results do not alter when nulliparous are or are not included (see Supplementary Results; Supplementary Tables 7–8).

### Modeling family planning influences on the distribution of fertility across the transition

To evaluate the influence of family planning in mediating changes in the distribution of fertility across the transition, we include a dummy variable in each of the hazard models described above, indicating whether a woman received a tubal ligation. While tubal ligation marks the end of a reproductive lifespan, this binary variable captures any difference between women who end up having a tubal ligation, and those who do not.

We assess the effects of tubal ligations on the distribution of fertility by testing for differences in all reproductive traits (mean age at first birth, age at last birth, and completed family size) using a non-parametric Mann–Whitney U test among women with and without a tubal ligation within a cohort. Further, we evaluate whether women receiving tubal ligations differ significantly in terms of the wealth, education, or market engagement compared to women who never received one (Table 4).

### Ethics statement

The Maya research has been approved by the Human Subjects IRB at Stony Brook University (20074791), Harvard University (F18643-101-104) and the University of Utah (00065740, 00093510), and by members and leaders of the Maya community. Informed consent was obtained from all subjects or, if subjects are under 18, from a parent and/or legal guardian.

## Supplementary Information

Supplementary Information

## Data Availability

To protect the privacy of the participants in this study, the data are not placed in a public depository. However, they are available by request to the corresponding author.

## Code availability

The R code that supports this study’s findings is available by request from the corresponding author.
