# Supplementary material for: Effects of family planning on fertility behaviour across the demographic transition
Source: Sci Rep. 2021 Apr 23;11:8835. doi: 10.1038/s41598-021-86180-8 (PMC8065026; doi:10.1038/s41598-021-86180-8)
Supplement: Supplementary file 1 — Supplementary Information [file 41598_2021_86180_MOESM1_ESM.docx]

**Supplementary Material**

**Does Family Planning Account for Fertility Behaviourn across the Demographic Transition?** Karen L Kramer, Joe Hackman, Ryan Schacht and Helen E. Davis

**Joe Hackman, November 2020**

The following describes the additional analyses and data presentations to supplement the main text. The supporting information is presented in three sections. Supplementary Methods extends our descriptives of the sample and variable construction. Supplementary Results presents supplemental analyses of key features of the data, including more detailed comparisons of reproductive traits and household characteristics across groups and cohorts. Supplementary Results also includes model results for the main model run on a restricted sample of ever-married women with no known fertility issues.

1. **Supplementary Methods**
   1. **Variable Construction**
   2. **Gini estimation**
   3. **Distribution of Fertility by Cohort**
   4. **Bootstrap test of homogeneity of variances**
2. **Supplementary Results**
   1. **Sharing Group Size**
   2. **Mean Differences in Fertility Variables by Cohort**
   3. **Mean Differences in Explanatory Variables by Cohort**
   4. **Bivariate Associations Between Total Fertility and Model Covariates**
   5. **Alternative Model Specifications**
      1. **Alternative coding of Wage-Labor**
      2. **Model on Marital Fertility**
   6. **Mean Comparisons of TL and Non-TL Women**

**Supplementary Methods**

**Variable Construction**

*Hectares under cultivation:* Land tenure was captured at the sharing-group level. The model variable was calculated as the total number of hectares of all land under cultivation by the sharing group, divided by the total number of adults (age 15>) living in the household. This land per-capita estimate accounts for any covariance between sharing group size and sharing group land holdings.

*Sharing Group Size:* Individuals live in household compounds called *solars*, which often include a patriarch and /or matriarch and their adult children’s families. These typically correspond to how households pool labor and share resources. However, in some instances, solars contain multiple families that do not identify as pooling labor and resources. Sharing group captures the extent of labor and resource pooling and typically maps onto geographic distributions of extended families. The variable used in the model is the total number of adults (age 15 >) living in the sharing group.

*Education:* This captures the total number of years of schooling of the focal woman.

*Wage-Labor:* Given most households engage in some form of wage-labor or another over the course of season, we chose to create a variable that captured when a household has strongly committed to wage-labor as a primary means of subsistence. This binary variable is 0 when the proportion of adults in the household engaged in wage labor is less than 50% and 1 when 50% or more of the adults identified wage-labor as a primary economic activity.

**Gini Estimation of Wealth and Fertility**

Changes in fertility after the introduction of the road coincide with increasing inequality along the primary measures of wealth within the community (Supplementary Table 1). As noted in the main text, prior to the completion of the road, inequality across resource (land and labor) as well as reproductive inequality was relatively low (Gini Coefficients =0.2, 0.21, and 0.19 respectively), compared to average estimated coefficients for a cross-cultural sample of agricultural population (Material Wealth Gini~0.4-0.5) ^65^.

| Supplementary Table 1. Mean, SD, and Gini Coefficients | | | | | | | | | | | |
| --- | --- | --- | --- | --- | --- | --- | --- | --- | --- | --- | --- |
|  | Hectares | | |  | Sharing Group Size | | |  | Total Fertility | | |
| Cohort | Mean | SD | Gini |  | Mean | SD | Gini |  | Mean | SD | Gini |
| 0 | 2.92 | 1.03 | 0.2 |  | 4.31 | 1.62 | 0.21 |  | 6.84 | 2.45 | 0.19 |
| 1 | 5.75 | 2.96 | 0.27 |  | 5.47 | 2.72 | 0.27 |  | 5.94 | 3.5 | 0.32 |
| 2 | 7.1 | 3.33 | 0.26 |  | 4.55 | 1.99 | 0.24 |  | 3 | 2.51 | 0.45 |
| 3 | 9.07 | 8.77 | 0.46 |  | 4.89 | 2.62 | 0.29 |  | 1.74 | 1.51 | 0.48 |
|  | | | | | | | | | | | |

**Supplementary Fig 1. Distribution of Fertility by Cohort.** Graph depicts the shifting distribution of fertility by 10-year cohort. The overlapping density plots shows the shift in means relative to other cohorts with expanding variance among completed fertility of women born in the 1960s.


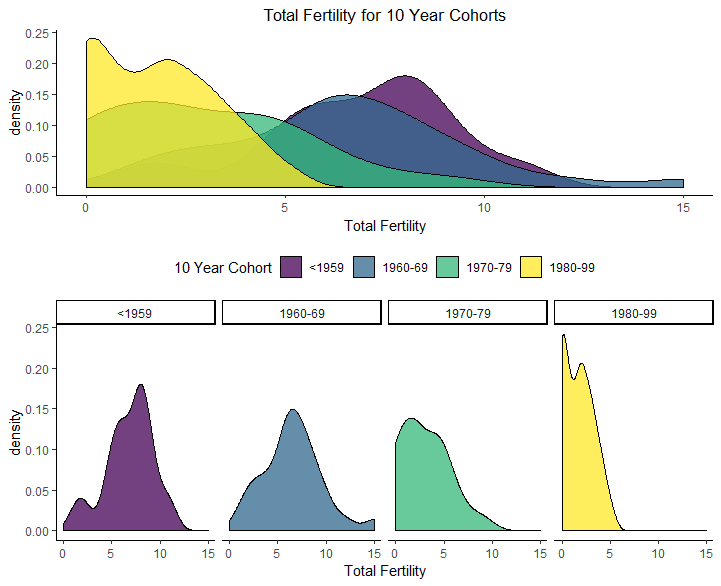


**Bootstrap Test for Homogeneity of Variances**

The manuscript uses the Coefficient of Variation to test for differences in fertility variance across cohorts. This measure of variance is standardized by the mean so it is comparable across cohorts when the mean fertility values change. To further assess whether the observed differences in fertility variance are not driven by small sample sizes across cohorts, we also employ a bootstrap test for homogeneity of variances on the unstandardized fertility variances (Cahoy 2010), using the testequavar package in R. The results do not return a p-value but rather an inference of whether the null hypothesis of equal variances can be rejected. Using 1,000 bootstrap samples, the test results are presented below.

- Cohort 0 – Cohort 1: Reject the Null – Variances are significantly different
- Cohort 1 – Cohort 2: Failed to Reject the Null – Variances are not significantly different
- Cohort 0 – Cohort 2: Failed to Reject the Null – Variances are not significantly different

Cahoy, Dexter O. "A bootstrap test for equality of variances." *Computational statistics & data analysis* 54.10 (2010): 2306-2316.

**Supplementary Results**

**Mean Differences in Fertility Variables by Cohort**

Mean comparison tests of reproductive variables by cohort are presented in Supplementary Table 3. The adjusted tests were used to assess significance of differences presented in Fig. 2 in the main manuscript.

| Supplementary Table 2. Dunn's Kruskal-Wallis Multiple Comparisons | | | |
| --- | --- | --- | --- |
| Age at First Birth | | | |
| Cohort Comparison | Z | P.unadj | P.adj |
| <1959 - 1960-69 | 1.28 | 0.202 | 0.4043 |
| <1959 - 1970-79 | -1.15 | 0.252 | 0.2520 |
| 1960-69 - 1970-79 | -2.25 | 0.025 | 0.0741 |
|  |  |  |  |
| Age at Last Birth | | | |
| Comparison | Z | P.unadj | P.adj |
| <1959 - 1960-69 | 1.90 | 0.057 | 0.1149 |
| <1959 - 1970-79 | 3.44 | 0.001 | 0.0017 |
| 1960-69 - 1970-79 | 1.80 | 0.071 | 0.0711 |
|  |  |  |  |
| Inter-Birth Intervals | | | |
| Comparison | Z | P.unadj | P.adj |
| <1959 - 1960-69 | 0.81 | 0.419 | 0.8378 |
| <1959 - 1970-79 | 1.29 | 0.196 | 0.5894 |
| 1960-69 - 1970-79 | 0.59 | 0.552 | 0.5522 |
|  |  |  |  |
|  |  |  |  |
| Completed Fertility | | | |
| Comparison | Z | P.unadj | P.adj |
| <1959 - 1960-69 | 1.19 | 0.235 | 0.2354 |
| <1959 - 1970-79 | 4.37 | 0.000 | 0.0000 |
| 1960-69 - 1970-79 | 3.34 | 0.001 | 0.0017 |
|  |  |  |  |

**Mean Differences in Explanatory Variables by Cohort**

Mean comparison tests of traditional and novel determinants by cohorts are presented in Supplementary Table 4. The results show increases in mean land and years of education across cohorts, while proportion of wage labor jobs and sharing group size has remained relatively constant.

| Supplementary Table 3. Dunn's Kruskal-Wallis Multiple Comparisons | | | |
| --- | --- | --- | --- |
| Hectares under cultivation | | | |
| Cohort Comparison | Z | P.unadj | P.adj |
| <1959 - 1960-69 | -4.04 | 0.000 | 0.0001 |
| <1959 - 1970-79 | -4.45 | 0.000 | 0.0000 |
| 1960-69 - 1970-79 | -0.97 | 0.334 | 0.3338 |
|  |  |  |  |
| Sharing Group Size | | | |
| Comparison | Z | P.unadj | P.adj |
| <1959 - 1960-69 | -1.67 | 0.094 | 0.2828 |
| <1959 - 1970-79 | 0.20 | 0.842 | 0.8425 |
| 1960-69 - 1970-79 | 1.64 | 0.101 | 0.2011 |
|  |  |  |  |
| Years of Education | | | |
| Comparison | Z | P.unadj | P.adj |
| <1959 - 1960-69 | -3.83 | 0.000 | 0.0003 |
| <1959 - 1970-79 | -4.04 | 0.000 | 0.0002 |
| 1960-69 - 1970-79 | -0.74 | 0.461 | 0.4614 |
|  |  |  |  |
| Proportion of Wage Laborers | | | |
| Comparison | Z | P.unadj | P.adj |
| <1959 - 1960-69 | 1.61 | 0.107 | 0.3204 |
| <1959 - 1970-79 | 0.80 | 0.423 | 0.8452 |
| 1960-69 - 1970-79 | -0.60 | 0.545 | 0.5452 |
|  |  |  |  |

**Supplementary Fig 2. Bivariate Associations Between Total Fertility and Model Covariates.** Graph shows positive effects of raw measures of sharing group and education, and to a lesser extent land on fertility in Cohort 1. Reproduction in Cohort 2 was characterized by the widespread adoption of Tubal Ligation, and modelling showed no significant effects of any predictors on fertility. By Cohort 3, land, education, and sharing group size all showed significant negative associations with fertility.


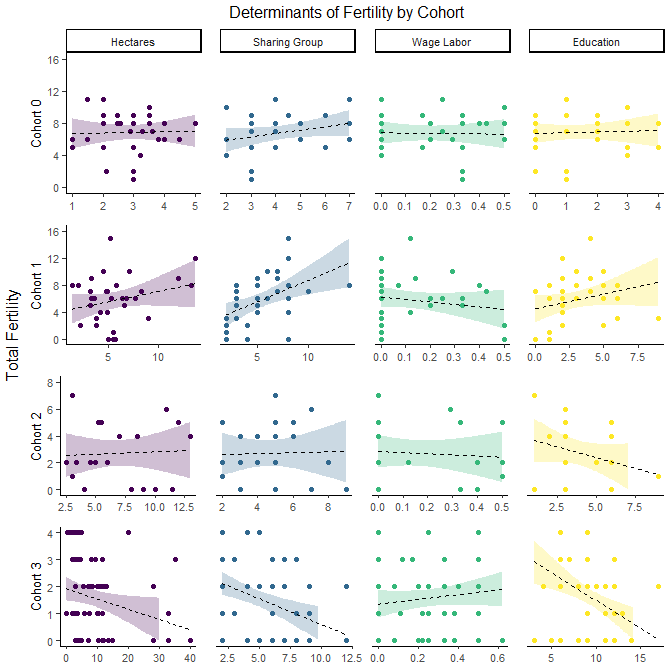


**Models with alternative coding of engagement in wage-labor**

Here we present model results using an alternative coding for household engagement in wage-labor. Rather than dummy coding households that are highly committed to wage labor, we include the proportion of adults in the household classified as wage-laborers as a continuous variable, and an alternate dummy variable indicating if any adult in the household was classified as a wage-laborer. Models with alternative coding for household engagement in wage labor are presented in Supplementary Table 5 and Supplementary Table 6. Using a binary variable indicating if any household member was coded as a wage laborer, or a quantitative variable coding proportion of the adults in the household engaged in wage labor did not qualitatively change the results.

| Supplementary Table 4. Hazard Ratios (95% CI) - Any Wage Laborer | | | | |
| --- | --- | --- | --- | --- |
|  | Cohort 0 | Cohort 1 | Cohort 2 | Cohort 3 |
| Hectares | 1.00 (0.92, 1.07) | 0.99 (0.96, 1.03) | 1.01 (0.95, 1.07) | 1.00 (1.00, 1.02) |
| Sharing Group Size | **1.06* (1.00, 1.12)** | **1.05*** (1.02, 1.07)** | 1.02 (0.88, 1.18) | **0.95* (0.88, 0.99)** |
| Years of Education | **1.08* (1.02, 1.15)** | **0.92* (0.86, 0.99)** | 0.94 (0.81, 1.09) | **0.96* (0.87, 0.98)** |
| Wage-Labor | 1.10 (0.92, 1.30) | 0.84 (0.70, 1.01) | 1.16 (0.67, 2.01) | 0.92 (0.67, 1.13) |
| Tubal Ligation | NA | 1.10 (0.92, 1.31) | NA | 1.28 (1.10, 1.76) |
| Observations | 235 | 205 | 87 | 199 |
| Wald Test | 14.72** (df = 4) | 17.66** (df = 5) | 1.37 (df = 4) | 21.39*** (df = 5) |
| *p<0.05 **p<0.01 ***p<0.001 | | | | |

| Supplementary Table 5. Hazard Ratios (95% CI) - Proportion of HH Wage Laborers | | | | |
| --- | --- | --- | --- | --- |
|  | Cohort 0 | Cohort 1 | Cohort 2 | Cohort 3 |
| Hectares | 1.01 (0.93, 1.09) | 1.00 (0.96, 1.03) | 1.01 (0.95, 1.08) | 1.00 (1.00, 1.02) |
| Sharing Group Size | 1.06 (1.00, 1.13) | **1.04** (1.01, 1.06)** | 1.03 (0.89, 1.19) | **0.93* (0.86, 0.99**) |
| Years of Education | **1.09* (1.02, 1.16)** | **0.92* (0.86, 0.99)** | 0.94 (0.81, 1.09) | 0.97 (0.88, 0.99) |
| Wage-Labor | 1.01 (0.58, 1.78) | 0.70 (0.38, 1.28) | 1.11 (0.31, 4.00) | **0.53* (0.28, 1.13)** |
| Tubal Ligation | NA | 1.08 (0.90, 1.30) | NA | **1.32* (1.12, 1.78)** |
| Observations | 235 | 205 | 87 | 199 |
| Wald Test | 14.03** (df = 4) | 12.07* (df = 5) | 1.23 (df = 4) | 25.21*** (df = 5) |
| *p<0.05 **p<0.01 ***p<0.001 | | | | |

**Models excluding nulliparous women and women with known fertility issues**

Here we re-run the main analysis on a restricted subset of the women that excludes nulliparous women, or women with known fertility issues. Supplementary Table 7 reports the exclusion decision and the final sample size for these alternative models. The results of the models are presented in Supplementary Table 8. The results are qualitatively similar to those presented in the main analyses. With these exclusions, in Cohort 2 there are no households where 50% or more of the adults are engaged in wage labor. We include an alternative model with a continuous variable for wage-labor coding. The results are presented in Supplementary Table 9 and again are qualitatively similar to results presented in the main analyses. The effect of wage-labor in Cohort 3 is reduced and non-significant. This is likely the result of excluding the large number of unmarried nulliparous women from analyses as they comprise a large proportion of the sample in Cohort 3 (~38%).

| Supplementary Table 6. Sample Exclusions by Cohort | | | | | |
| --- | --- | --- | --- | --- | --- |
| Cohort | All Women | Excluded | % of Cohort | Final Sample | (n) – Reasons |
| <1959 | 31 | 6 | 23% | 27 | (2) - Unmarried; (2) - Uncertain fertility |
| 1960-69 | 33 | 6 | 18% | 27 | (4) - No Kids; (1) - One child, fertility issues |
| 1970-79 | 22 | 6 | 27% | 16 | (6) - No Children |
| 1980-89 | 32 | 5 | 16% | 27 | (5) - Unmarried / No Children |
| 1990-99 | 45 | 17 | 38% | 28 | (17) - Unmarried / No Children |

| Supplementary Table 7. Main Model Excluding nulliparous women. Hazard Ratios (95% CI) | | | | |
| --- | --- | --- | --- | --- |
|  | Cohort 0 | Cohort 1 | Cohort 2 | Cohort 3 |
| Hectares | 1.02 (0.94, 1.11) | 0.99 (0.96, 1.03) | 1.02 (0.95, 1.09) | 0.99 (0.98, 1.02) |
| Sharing Group Size | 1.03 (0.97, 1.10) | **1.04** (1.01, 1.06)** | 1.13 (0.98, 1.31) | **0.89*** (0.82, 0.94)** |
| Years of Education | **1.11** (1.04, 1.18)** | **0.92* (0.86, 0.99)** | 1.02 (0.93, 1.12) | 0.98 (0.92, 0.98) |
| Wage-Labor | 0.76 (0.53, 1.10) | NA | 1.28 (0.69, 2.37) | **0.54*** (0.42, 0.84)** |
| Tubal Ligation | NA | 1.04 (0.86, 1.25) | NA | **1.32** (1.11, 1.66)** |
| Observations | 232 | 202 | 80 | 195 |
| Wald Test | 17.98** (df = 4) | 13.09* (df = 4) | 5.99 (df = 4) | 37.36*** (df = 5) |
| *p<0.05 **p<0.01 ***p<0.001 | | | | |

| Supplementary Table 8. Main Model Excluding nulliparous women and alternative wage-labor coding. Hazard Ratios (95% CI) | | | | |
| --- | --- | --- | --- | --- |
|  | Cohort 0 | Cohort 1 | Cohort 2 | Cohort 3 |
| Hectares | 0.99 (0.92, 1.07) | 0.99 (0.95, 1.02) | 1.02 (0.94, 1.09) | 1.00 (0.99, 1.03) |
| Sharing Group Size | 1.06 (1.00, 1.12) | **1.05*** (1.02, 1.07)** | 1.10 (0.97, 1.23) | **0.95* (0.88, 0.98)** |
| Years of Education | **1.08* (1.01, 1.15)** | **0.92* (0.86, 0.98)** | 1.03 (0.93, 1.14) | **0.96 *(0.88, 0.98)** |
| Wage-Labor | 1.10 (0.92, 1.31) | 0.85 (0.71, 1.01) | 1.15 (0.65, 2.03) | 0.86 (0.63, 1.04) |
| Tubal Ligation | NA | 1.08 (0.90, 1.29) | NA | **1.26 **(1.08, 1.72)** |
| Observations | 232 | 202 | 80 | 195 |
| Wald Test | 13.73^**^ (df = 4) | 16.05^**^ (df = 5) | 7.87 (df = 4) | 23.05^***^ (df = 5) |
| *p<0.05 **p<0.01 ***p<0.001 | | | | |

**Mean comparison of TL and Non-TL women in Cohorts 1 and 3**

To further test for an effect of TL on changes in completed family size among women across each cohort, we compared TL and Non-TL women’s reproductive traits and socioeconomic variables using a non-parametric Mann-Whitney U-test. Supplementary Table 10 reports the mean comparison tests between TL and Non-TL women for cohorts 1 and 3. Results show no significant differences between TL and Non-TL women in Cohort 1, the first cohort following the completion of the road. Again, early adopters of TL did not use family planning to significantly limit completed family size. However, by Cohort 3, TL women had significantly lower family sizes and significantly lower age at last birth. Additionally, TL adoption is more frequent among wealthier, more educated women.

| Supplementary Table 9. Comparison of Women with and without tubal ligations. | | | | | | | | |
| --- | --- | --- | --- | --- | --- | --- | --- | --- |
|  |  | Cohort 1 | | |  | Cohort 3 | | |
|  |  | Non-Tubal Ligation (N=12) | Tubal Ligation (N=17) | P-value |  | Non-Tubal Ligation (N=41) | Tubal Ligation (N=12) | P-value |
| *Demographic Traits* |  |  |  |  |  |  |  |  |
| Age at First Birth |  | 22.1 (6.1) | 19.9 (3.7) | 0.28 |  | 21 (3.2) | 20 (3.2) | 0.34 |
| Age at Last Birth |  | 34 (4.8) | 35.6 (5) | 0.38 |  | 25.3 (4) | 26.1 (2.3) | 0.38 |
| Completed Fertility |  | 4.9 (4) | 6.8 (2.8) | 1.40 |  | **2.3 (1.1)** | **3.2 (1)** | **0.02** |
| Age |  | -- | -- |  |  | **27.3 (4.5)** | **31.7 (2.5)** | **<0.001** |
| *Socioeconomic* |  |  |  |  |  |  |  |  |
| Hectares of Land |  | 6.2 (3.2) | 5.3 (2.8) | 0.43 |  | **8.3 (8.1)** | **5.2 (3)** | **0.05** |
| Years of Education |  | 2.5 (1.8) | 3.4 (2.3) | 0.21 |  | **9.4 (2.6)** | **7.9 (1.7)** | **0.03** |
| Sharing Group Size |  | 5.2 (2.3) | 5.7 (3.1) | 0.60 |  | 4.5 (2.6) | 4.4 (2.4) | 0.90 |
| Proportion of Wage Labor |  | 0.1 (0.2) | 0.1 (0.2) | 0.90 |  | 0.2 (0.2) | 0.3 (0.2) | 0.40 |
| *Notes: Does not include comparisons for Cohort 2 because 16 out of 17 women who had children received a Tubal Ligation. Comparisons excludes nulliparous women since they do not tubal ligations. | | | | | | | | |
